# Construction and evaluation of an integrated “Hospital-Community-Family” public cardiopulmonary resuscitation training system

**DOI:** 10.3389/fpubh.2025.1541177

**Published:** 2025-06-11

**Authors:** Yanhua An, Yun Wei, Dawei Wang, Bingchen Ma, Hua Wang, Qiumei Cao

**Affiliations:** ^1^Department of General Practice, Beijing Tongren Hospital, Capital Medical University, Beijing, China; ^2^Emergency Department, Beijing Tongren Hospital, Capital Medical University, Beijing, China

**Keywords:** general practitioners, family caregivers, cardiopulmonary resuscitation (CPR), training, feasibility

## Abstract

**Background:**

Epidemiological investigations have found that 80% of cardiac arrest (CA) events occur in public places or homes. Bystander cardiopulmonary resuscitation (CPR) is the most significant factor for out-of-hospital CA (OHCA) survival. Limited opportunities exist for community residents and family members of patients with chronic diseases to participate in CPR training programs. This study establishes an integrated “Hospital-Community-Family” public CPR training system and assesses its training feasibility.

**Methods:**

Training system construction: the integrated “Hospital-Community-Family” public CPR training system included three levels members and two steps. The three levels members were emergency physicians at level A tertiary hospital, general practitioners (GPs) from community health service centers, and family members of patients with chronic diseases. Two steps included: (1) GPs receiving public CPR training by emergency physicians, passing the examination, and obtaining American Heart Association (AHA) certificate; (2) family members of patients with chronic diseases receiving CPR training from GPs in community health service centers. Training feasibility assessment: a questionnaire survey was used to investigate the CPR knowledge and cognition of family members of chronic disease patients before, after and 6 months after training.

**Results:**

Construction of the integrated “Hospital-Community-Family” public CPR training system involved eight trainers certified in American Heart Association (AHA) CPR training from the level A tertiary hospital, 23 general practitioners from the community who completed the AHA training and obtained certificates, and 149 family members of patients with chronic diseases under community care who received training. Training feasibility assessment was conducted of family members before, immediately after and 6 months post training, yielding mean scores of 9.83 ± 4.11, 13.97 ± 2.87, and 13.02 ± 3.12 (out of a total score of 17), respectively. The differences were statistically significant (*p* < 0.001). After 6 months, nearly half of the family members of patients with chronic diseases believed they possessed adequate CPR knowledge and skills and were confident in their CPR abilities.

**Conclusions:**

The proposed integrated “Hospital-Community-Family” public CPR training system demonstrated significant acceptability, practical feasibility, and the necessity for its implementation.

## 1 Introduction

Sudden cardiac death (SCD) refers to a natural death caused by various cardiac reasons. It occurs suddenly, progresses rapidly, and fatality occurs within 1 h of symptom onset. SCD accounts for 18% of all natural deaths and 50% of deaths from coronary atherosclerotic heart diseases (1). The annual incidence of SCD in the United States is 0.1–0.2% (2). The results of a national “Tenth Five-Year Plan” project led by Fuwai Hospital revealed that the annual incidence of SCD in China is 41.84 per 100,000 (0.04%) (3).

Studies indicate that effective cardiopulmonary resuscitation (CPR) within the first 4 min of sudden cardiac arrest (CA) can result in a rescue success rate of up to 50%. Each 1-min delay in CPR reduces the survival rate by 7–10%, and after 10 min, the success rate drops to < 1% (4). Globally, CA incidence ranges from 20 to 140 cases per 100,000 people, with a survival rate of only 2–11% (2). Developed countries prioritize the dissemination of public CPR techniques. In China, < 1% of the population is trained in CPR, whereas the figures are 33% for the United States and 40% for France. Correspondingly, the survival rate of out-of-hospital CA (OHCA) patients in China is < 1%, significantly lower than the 10–12% in Western countries (5, 6).

Historically, public CPR training in China has primarily focused on individuals in key sectors such as education, science and technology, public security, transportation, and service industries (7, 8). Limited opportunities exist for community residents and family members of patients with chronic diseases to participate in such training programs. Epidemiological investigations have found that 80% of CA events occur in public places or homes, with community residents often being the first witnesses to OHCA events (9). Bystander CPR is the most significant factor for OHCA survival. Studies have shown the effectiveness of bystander CPR was 30.5 vs. 16.6% without bystander CPR (10). Studies have shown the significance of CPR training for family members of patients with cardiac diseases (11).

During the 19th century, Ebbinghaus, a German psychologist, conducted a comprehensive investigation into the phenomenon of “forgetting,” determining that the process commences immediately following the initial acquisition of knowledge. In the context of CPR training, the issue of skill decay post-training also arises. Research in medical education suggests that skills related to CPR can deteriorate by 30–50% within 3–6 months, if no refresher training is provided (12). Previous CPR training sessions were conducted in a randomized manner, lacking continuity within the same demographic group (5). These studies suggest the importance of continuous skill reinforcement in CPR training.

This study aims to establish an integrated “Hospital-Community-Family” public CPR training system. The system will be led by emergency physicians from level A tertiary hospital as instructors, with GPs in community health service centers as trainers, and family members of patients with chronic diseases under community care as trainees. Additionally, we also evaluated the feasibility of the newly established system.

The studies involving human participants were reviewed and approved by the ethics committee of Beijing Tongren Hospital affiliated to Capital Medical University and the reference number is TRECKY2021-058. Written informed consent to participate in this study was provided by the participant.

## 2 Methods

### 2.1 Construction of the integrated “Hospital-Community-Family” CPR training system

#### 2.1.1 The structure of integrated “Hospital-Community-Family” CPR training system

AHA CPR instructors team: this team consists of eight emergency physicians from the level A tertiary hospital who hold American Heart Association (AHA) CPR instructor qualifications. The instructor team collaboratively developed a unified training program following the AHA Basic Life Support (BLS) Course. Two instructors formed a teaching unit, adhering to a 2:5 instructor-to-learner ratio for training community general practitioners (GPs).

Trainer team: this team consists of GPs from community health service centers. Using a blend of classroom teaching and simulated human training, the instructor team provided standardized training to GPs. After assessing their CPR performance, those who passed the assessment joined the training team. Two certified community trainers formed a teaching unit, maintaining a 2:5 ratio to train family caregivers.

Trainee team: this team comprised family members of patients with chronic diseases who are enrolled in the community health service system and reside near community hospitals. GPs provided CPR training to those family members in community health service centers, which involved classroom teaching and simulated human practice, followed by a CPR performance assessment.

Trainees inclusion criteria: (1) family members of patients with hypertension, coronary heart diseases, or diabetes. (2) Age ≥18 years or ≤ 70 years. (3) Living together with those patients. (4) Agreement to participate in this training.

Trainees exclusion criteria: (1) family members who have participated in CPR training within the past 2 years; (2) family members with implantable cardioverter defibrillators or with severe physical or mental illnesses.

#### 2.1.2 Training content

- Basic first aid concepts: this section covers principles of pre-hospital emergency care, emergency call knowledge (including China's 120 emergency system and telephone-assisted CPR protocols), identification of cardiac arrest, and the importance of first responder assistance.
- CPR skills: training incorporated both Hands-Only CPR (chest compressions) and traditional CPR with ventilation. For participants with no prior experience, we adopted a stepwise approach: initial demonstration of compression-only technique followed by integrated ventilation training using manikins.
- Psychological preparedness for on-site emergency response.
- Emergency system integration: special emphasis was placed on China's telephone-assisted CPR system where 120 dispatchers provide real-time guidance to callers, including compression rhythm coaching and emergency positioning through smartphone networks.

#### 2.1.3 Training materials

(1) Courseware: Unified PowerPoint slides based on the Chinese Public Health Guidelines for cardiopulmonary resuscitation. (2) Training manuals and videos: training content compiled into the CPR skills training manual, combined with CPR videos and standardized CPR operation videos recorded by instructors, was distributed to trainees. (3) Training equipment: CPR manikin, automated external defibrillator (AED), etc.

#### 2.1.4 Training process

Trainees participate in the training program in separate sessions, with each session accommodating five trainees and duration lasting 4 h.

- Theoretical training: trainers explained the importance of CPR, broke down CPR operation essentials step by step, demonstrated the CPR manikin, and concluded with a complete CPR operation video.
- Group practice: trainees practice the CPR manikin in groups, with demonstrations and guidance by trainers.
- Assessment: the standardized assessment protocol from the AHA Basic Life Support (BLS) Course was adopted, comprising both theory and skill evaluations.

The scoring scale designed based on the AHA Basic Life Support (BLS) Course for both theory and skills. The theory test consists of 10 questions with a total score of 10 points. Trainees with a score below 6 need to be re-examined.

The skill assessment includes identification and initiation of CPR, chest compressions, airway opening and ventilation, and AED usage. Each item on the assessment is scored on a scale of “not achieved,” “partially achieved,” “basically achieved,” and “fully achieved”, with scores ranging from 1 to 4. If two or more items are marked as “not achieved,” remediation is necessary. One “not achieved” equals two “partially achieved” and three “basically achieved”. The higher the score, the better the performance. All assessments were conducted using validated CPR training manikins with real-time feedback capabilities.

### 2.2 Evaluation of training feasibility

#### 2.2.1 Participants- family members of patients with chronic diseases

The family members of patients with chronic diseases from Majuqiao Community Health Service Center, Tiantan Community Health Service Center, Yizhuang Community Health Service Center, and Yinghai Community Health Service Center participated in this training and survey.

#### 2.2.2 Evaluation tools

A self-designed questionnaire based on literature about CPR was used in this study, including: (1) basic information; (2) cognization of CPR; (3) knowledge of CPR (including CPR timing, operational precautions, criteria for successful resuscitation, etc.); and (4) satisfaction of this training. The questionnaire was constructed through a series of group discussions to systematically validate each item. The team comprised two GPs with doctoral degree and four emergency physicians with master's degree.

#### 2.2.3 Evaluation method

An online questionnaire survey was conducted online (https://www.wjx.cn/) before training (August 2022), immediately after training (September 2022), and 6 months after training (March 2023) among family members of chronic disease patients.

#### 2.2.4 Statistical methods

Data from the online survey were exported and analyzed using SPSS 19.0. Descriptive statistics were represented as mean ± standard deviation for continuous variables, and frequency and percentages for categorical variables. Chi-square tests were employed for statistical inference for categorical variables, repeated measures analysis of variance was applied to compare three sets of continuous data, with a significance level set at *p* < 0.05.

## 3 Results

### 3.1 Results of the development of the integrated “Hospital-Community-Family” public CPR training system

The integrated “Hospital-Community-Family” public CPR training system was conducted, including:

- AHA CPR instructors: finally, eight emergency physicians in Beijing Tongren Hospital participated in this study as AHA CPR instructors.
- CPR trainers: in total, 23 GPs form four community health service centers (with five from Majuqiao Community Health Service Center, five from Tiantan Community Health Service Center, five from Yizhuang Community health Service Center, and eight from Yinghai Community Health Service Center) participated in this study, obtained AHA certificates, and be as CPR trainers.
- Family members of patients with chronic diseases: a total of 149 family members of patients with chronic diseases participated in CPR training in this study.

### 3.2 Results of training feasibility evaluation

#### 3.2.1 Basic information on family members of chronic disease patients

All 149 family members of patients with chronic diseases who participated in CPR training answered our survey online, with an average age of 48.8 ± 15.8 years. Among them, 73.2% were female and 85.2% were married. In addition, 40.9% had a university degree or higher, 54.4% had completed middle school education, and only 4.7% had education at primary school. Out of the participants, 84.6% were non-medical workers, 61.1% of the family members had no history of chronic diseases (Table 1).

**Table 1:** Basic information on family members of patients with chronic diseases.

**Item**	**Frequency**	**Percentage %**
**Gender**		
Male	40	26.8
Female	109	73.2
**Age**		
≤ 45 years	68	45.6
≤ 46–64 years	55	36.9
≥65 years	26	17.5
**Marital status**		
Unmarried	19	12.8
Married	127	85.2
Divorced	1	0.7
Widowed	2	1.3
**Education level**		
Elementary school or below	7	4.7
Junior high school	32	21.5
Senior high school/vocational school	49	32.9
University and above	61	40.9
**Occupation**		
Medical worker	23	15.4
Non-medical worker	126	84.6
**Annual family income (CNY, China Yuan; CNY/USD** = **0.14)**		
< 50,000	56	37.6
50,000–100,000	53	35.6
>100,000	40	26.8
**Physical condition (multiple choice)**		
No chronic diseases	91	61.1
Hypertension	42	28.2
Diabetes	24	16.1
Coronary heart disease	15	10.1
Cerebrovascular disease	9	6.0
Other	9	6.0

#### 3.2.2 Cardiovascular risk factors in participating family

Among the participating families, 63.8% had elevated blood pressure, 49.7% had elevated cholesterol levels, 44.3% had elevated low-density lipoprotein levels, 40.9% had elevated blood sugar, 54.4% were overweight or obese, and 22.1% were smokers.

#### 3.2.3 Knowledge of CPR among family members who participated in the training

Before the CPR training, 79.2% of participants had heard of CPR, and nearly half wish to receive a CPR training, but had no suitable training opportunities. Close to one-third were unaware of the significance of CPR, and 27.5% lacked time for CPR training. Approximately 12.1% had previously encountered situations requiring emergency rescue. In addition, 95.9% thought they needed CPR training, while 89.9% felt capable of learning CPR knowledge and skills. Moreover, 91.3% expressed willingness to perform CPR on someone experiencing sudden cessation of breathing and heartbeat after receiving training. However, 8.7% were reluctant to perform CPR owing concerns about insufficient emergency response ability (Table 2).

**Table 2:** Knowledge of CPR among family members who participated in the trainings.

**Item**	**Frequency**	**Percentage %**
**Heard of CPR before training**		
Yes	118	79.2
No	31	20.8
**Reasons for not having received CPR training previously**		
**(multiple choice)**		
Lack of awareness of its importance	43	28.9
Unable to find free training locations	76	51.0
Too busy with work or studies	41	27.5
No desire to attend training previously	4	2.7
Other	8	5.4
**Encountered situations requiring emergency rescue**		
Yes	18	12.1
No	131	87.9
**Necessity of learning CPR knowledge and skills for**		
**community residents**		
Not necessary at all	1	0.7
Not necessary	5	3.4
Necessary	40	26.8
Very necessary	103	69.1
**Perceived ability to learn CPR knowledge and skills**		
No ability at all	1	0.7
Limited ability	14	9.4
Some ability	76	51.0
High ability	58	38.9
**Willingness to perform CPR on someone experiencing sudden**		
**cessation of breathing and heartbeat after training**		
Unwilling	13	8.7
Worried about being misunderstood	4	2.7
Concerned about insufficient emergency response ability	8	5.4
Unwilling to touch someone close to death	1	0.7
Willing	136	91.3

#### 3.2.4 Feasibility of training

##### 3.2.4.1 Health literacy of family members of patients with chronic diseases before, after, and 6 months after training

A total of 149 family members of patients with chronic diseases participated in the CPR training. All respondents participated in pre-training, post-training surveys. Among them, 140 individuals participated in the 6-month follow-up survey, resulting in a follow-up loss rate of 6%. The mean scores of health literacy of family members of patients with chronic diseases in CPR were 9.83 ± 4.11 before training, 13.97 ± 2.87 after training, and 13.02 ± 3.12 6 months after training (total score: 17). The differences were statistically significant (*p* < 0.001) (Table 3) for details.

**Table 3:** Health literacy of family members of patients with chronic diseases before, after, and 6 months after training.

**Time of assessment**	***N***	**Mean ±standard deviation**	***F***	***p*-Value**
Before training	149	9.83 ± 4.11	59.62	< 0.001
After training	149	13.97 ± 2.87		
Six months after training	140	13.02 ± 3.12		

##### 3.2.4.2 Self-evaluation of CPR skills by family members of patients with chronic diseases after training

A total of 140 participants completed the follow-up survey 6 months after training. Regarding their CPR knowledge, 45.7% believed they had a good grasp, 46.4% rated their grasp as moderate, and only 7.1% felt their grasp was poor. In terms of skills, 42.1% believed they had good CPR skills, 47.1% assessed their skills as moderate, and only 10.7% considered their skills poor. Regarding confidence, 43.6% expressed confidence in their ability to perform CPR, 45.7% had moderate confidence, and only 10.7% lacked confidence. Additionally, 84.3% believed it was necessary to reinforce CPR training (Table 4) for details.

**Table 4:** Self-evaluation of CPR skills by family members of patients with chronic diseases after training (*n* = 140).

**Item**	**Frequency**	**Percentage %**
**How would you rate your grasp of CPR knowledge 6 months**		
**after training?**		
Very poor	3	2.1
Poor	7	5.0
Moderate	65	46.4
Good	36	25.7
Very good	29	20.7
**How would you rate your CPR skill level 6 months after**		
**training?**		
Very poor	2	1.4
Poor	13	9.3
Moderate	66	47.1
Good	29	20.7
Very good	30	21.4
**How would you rate your confidence in performing CPR**		
**6 months after training?**		
Very unconfident	2	1.4
Unconfident	13	9.3
Moderate	64	45.7
Confident	39	27.9
Very confident	22	15.7
**Do you think it is necessary to reinforce CPR training in**		
**the future?**		
Not necessary at all	2	1.4
Not necessary	0	0
Moderate	20	14.3
Necessary	84	60.0
Very necessary	34	24.3

##### 3.2.4.3 CPR implementation within 6 months after training

Within 6 months after training, 10 individuals (7.1%) performed CPR. The relationships between the rescuers and the victims were as follows: family members (one person), friends (three people), and strangers (6 people). During the CPR process, two individuals took on the role of the primary rescuer, five assisted others in performing CPR, and one guided someone else in performing CPR. Ultimately, two cases of CPR were successful (Table 5) for details.

**Table 5:** CPR implementation within 6 months after training (*n* = 140).

**Item**	**Frequency**	**Percentage %**
**Did you perform CPR after training?**		
Yes	10	7.1
No	130	92.9
**Relationship between the CPR rescuer and the victim**		
Family member	1	0.7
Friend	3	2.1
Stranger	6	4.2
**Role played during CPR**		
Primary CPR performer	2	1.4
Assistant, collaborated with others in CPR	5	3.6
Guided others in performing CPR	1	0.7
Other	2	1.4
**CPR results**		
Successful	2	1.4
Unsuccessful	8	5.8

##### 3.2.4.4 Evaluation of CPR training by family members of chronic disease patients

Regarding the CPR training, 92.8% expressed satisfaction with the course design, 95.0% were satisfied with the instructors, 95.7% were satisfied with the training content, and 94.3% were satisfied with the training methods. The majority of family members of chronic disease patients were overall satisfied with the CPR training (96.4%) and would recommend others to participate in the next CPR training session (97.1%; Table 6).

**Table 6:** Satisfaction with CPR training among family members of patients with chronic diseases (*n* = 140).

**Item**	**Frequency**	**Percentage %**
**How satisfied are you with the course design of this**		
**CPR training?**		
Very dissatisfied	6	4.3
Somewhat dissatisfied	0	0
Neutral	4	2.9
Satisfied	59	42.1
Very satisfied	71	50.7
**How satisfied are you with the instructors in this CPR training?**		
Very dissatisfied	5	3.6
Somewhat dissatisfied	0	0
Neutral	2	1.4
Satisfied	55	39.3
Very satisfied	78	55.7
**How satisfied are you with the content of this CPR training?**		
Very dissatisfied	5	3.6
Somewhat dissatisfied	0	0
Neutral	1	0.7
Satisfied	62	44.3
Very satisfied	72	51.4
**How satisfied are you with the methods of this CPR training?**		
Very dissatisfied	5	3.6
Somewhat dissatisfied	0	0
Neutral	3	2.1
Satisfied	61	43.6
Very satisfied	71	50.7
**How satisfied are you with the overall CPR training?**		
Very dissatisfied	3	2.1
Somewhat dissatisfied	0	0
Neutral	2	1.4
Satisfied	62	44.3
Very satisfied	73	52.1
**Would you recommend others to participate in future CPR**		
**training sessions?**		
Very unlikely	3	2.1
Somewhat unlikely	0	0
Neutral	1	0.7
Likely	66	47.1
Very likely	70	50.0

## 4 Discussion

This study established an integrated “Hospital-Community-Family” public CPR training system centered around Beijing Tongren Hospital, CMU, as well as four community health service organizations in the medical consortium affiliated to that hospital. Bystander CPR is the most significant factor for OHCA survival. As trainees, these family members often serve as the first witnesses to sudden cardiac arrests among patients with chronic diseases. Previous studies selected medical students (7) and college students (8) as trainees. This study chose family members of patients with chronic diseases as participants. Within 6 months after training, 10 trainees performed CPR, two of them were successful, demonstrating the rationale for selecting family members as trainees.

Anderson et al. (13) identified that high-frequency CPR training would improve the quality of bystander CPR skills. The shortage of comprehensive training and support systems in China leads to a dearth of instructors consistently engaged in teaching, while those trained through short-term programs lack a guarantee of teaching quality, ultimately impacting the training outcomes (14). In this study, nearly half of the trainees were unable to find suitable opportunities for training despite wanting to. In our study, community physicians and residents are engaged through contractual agreements to deliver services. General practitioners from the community conducted training, while family members of patients with chronic diseases under community care received training. The trainers' working environment and the trainees' living environment, as part of community care, exhibit a relatively stable nature, thereby augmenting the potential for consistent training sessions.

Presently, CPR training methods in China include traditional lecture-based instruction, multimedia training, human model-based teaching, scenario-based training, and online platforms like WeChat or micro-courses (15, 16). The proposed integrated “Hospital-Community-Family” CPR training system, with general practitioners from the community as the main trainers, possesses distinct advantages in terms of objective patient enrollment, stable personnel, and extended family management. This strategy contributes to the successful implementation, promotion, and continuity of CPR training, enhancing its effects in the long run.

This CPR training was evaluated for CPR-related health literacy among family members of patients with chronic diseases before training, immediately after training, and 6 months after training. The mean scores were 9.83 ± 4.11, 13.97 ± 2.87, and 13.02 ± 3.12, respectively, on a total score of 17, showing statistically significant differences. This indicates a substantial improvement in CPR-related knowledge and literacy among family members after training, with the acquired knowledge and skills being sustained 6 months post-training.

Recent studies (17) have found that emotional aspects become increasingly important in bystanders CPR performing, such as self-confidence and willingness to help. In this study, nearly half of the participants perceived their mastery of CPR knowledge and skills as good, and they were confident in their CPR abilities 6 months after training. Within the 6-month post-training period, these family members actively participated in CPR scenarios and demonstrated successful CPR in two instances.

The majority of family members expressed overall satisfaction with the CPR training program and were willing to recommend others to partake in subsequent CPR training sessions. These results underscore the significant impact and practical feasibility of the integrated “Hospital-Community-Family” CPR training system.

One limitation of this study is that the follow-up was conducted through surveys, without assessing their CPR skills. Previous research has shown a decline in CPR performance among healthcare workers after 6 months, highlighting the need for retraining. Implementing retraining in community settings may be more challenging, but future studies could explore ways to address this issue.

Furthermore, this study solely assessed the CPR practice of family members of patients with chronic diseases 6 months after training. Subsequent research can perform continued follow-ups on their CPR practices and gather more feedback from these family members regarding their CPR experiences. This feedback could serve as a reference for enhancing training effectiveness in the future.

The responses of family members are crucial, prompting the development of a CPR training program for them. Moving forward, there is potential to scale this model by incorporating more trainers, evaluating the frequency of necessary training for trainers, and identifying areas for further improvement. The implementation of a Contracted Family Doctor Service enhances the stability of the training system, increases its accuracy, ease of application, and functionality, and will improve the survival rate of OHCA cases.

## 5 Conclusions

The proposed integrated “Hospital-Community-Family” CPR training system demonstrated significant acceptability, practical feasibility, and the need for wider implementation.

## Data Availability

The raw data supporting the conclusions of this article will be made available by the authors, without undue reservation.
