# Supplementary material for: Construction and evaluation of an integrated “Hospital-Community-Family” public cardiopulmonary resuscitation training system
Source: Front Public Health. 2025 Jun 11;13:1541177. doi: 10.3389/fpubh.2025.1541177 (PMC12187777; doi:10.3389/fpubh.2025.1541177)
Supplement: Supplementary file 1 [file Table_1.pdf]

**Questionnaire on health literacy related to cardiopulmonary resuscitation**

(Before training)

In order to actively mobilize the family members of patients with chronic diseases to participate in pre-hospital first aid, so that family members can effectively play the role of on-site first aid as the first witnesses, this project provides CPR knowledge and skills training to family members of chronic disease patients in community contracted families. In order to evaluate the effectiveness of the program and make the training of family health workers more targeted, we will conduct a survey on the CPR related situation of family members, and we will keep your personal information strictly confidential, please ensure the authenticity of the information filled in.

Research group of general hospitals and community health service systems cooperating to build a CPR training system for high-risk patients with sudden death.

August 6, 2021

Community Healthcare Center: ______________

1. Basic information

1.1 Name: _______________

1.2 Gender:

○①Male

○②Female

1.3 Age: ________

1.4 Education level:

○①Elementary school or below

○②Junior high school

○③Senior high school/ vocational school

○④University and above

1.5 Occupation:

○①Medical worker

○②Non-medical worker:

1.6 Annual family income (CNY, China Yuan; CNY/USD=0.14):

○①<50,000

○②50,000-100,000

○③>100,000

1.7 Marital status:

○①Unmarried

○②Married

○③Divorced

○④Widowed

1.8 Physical condition (multiple choice):

○①No chronic diseases

○②Hypertension

○③Diabetes

○④Coronary heart disease

○⑤Cerebrovascular disease

○⑥Mental disease

○⑦Other:

1.9 Do you have a family member who has got cardiovascular disease?

○①Yes

○②No

1.10 Whether there are any of the following risk factors for cardiovascular disease in the family members:

1.10.1 Elevated blood pressure（≥ 140/90 mmHg）

○①Yes

○②No

1.10.2 Elevated total cholesterol (TC) level（≥ 4.5 mmol/L）

○①Yes

○②No

1.10.3 Elevated low-density lipoprotein cholesterol（LDL-C）level（⩾ 2.6 mmol/L）

○①Yes

○②No

1.10.4 Lowered high-density lipoprotein cholesterol（HDL-C）level（< 1.0 mmol/L）

○①Yes

○②No

1.10.5 Elevated fasting blood glucose（⩾ 7.0 mmol/L）

○①Yes

○②No

1.10.6 Overweight or obese（BMI ⩾ 24 $kg/m^2$）

○①Yes

○②No

1.10.7 Tobacco use

○①Yes: ___

○②No

2. Knowledge of CPR among family members who participates in the trainings

2.1 Have you heard of CPR before this survey?

○①Yes

○②No

2.2 Have you been trained in CPR within the past 2 years?

○①No

○②Yes, I was trained in CPR within the past 2 years.

2.3 Reasons for not having received CPR training previously (multiple choice).

□①Lack of awareness of its importance

□②Unable to find free training locations

□③Too busy with work or studies

☐④No desire to attend training previously

☐⑤Other:

2.4 How would you prefer to learn CPR?

○①On-site professional guidance

○②Video/audio learning

○③Learning via TV

○④Classroom-based learning

2.5 Have you encountered situations requiring emergency rescue?

○①No

○②Yes, encountered before

2.6 Do you think it is possible for patients with respiratory and/or cardiac arrest to be resuscitated?

○①Completely impossible

○②Generally impossible

○③Somewhat possible

○④Very possible

2.7 Necessity of learning CPR knowledge and skills for community residents.

○①Not necessary at all

○②Not necessary

○③Necessary

○④Very necessary

2.8 Do you believe you have the ability to learn CPR knowledge and skills?

○①Completely incapable

○②Generally incapable

○③Somewhat capable

○④Very capable

2.9 Do you have any interest in learning CPR knowledge and skills?

○① Completely uninterested

○② Generally uninterested

○③ Somewhat interested

○④ Very interested

2.10 Are you interested in participating in CPR training?

○ ①Completely uninterested

○ ②Generally uninterested

○ ③Somewhat interested

○ ④Very interested

2.11 If you were trained and mastered CPR, would you be willing to perform CPR on a family member or friend experiencing sudden cardiac arrest? (If you select "Unwilling," please proceed to Question 2.12)

○① Completely unwilling

○② Generally unwilling

○③ Somewhat willing

○④ Very willing

2.12 What is the primary reason for your unwillingness to perform CPR? (If you selected "Willing" in Question 2.11, skip this question)

○① Find it unsanitary

○② Fear of contracting an infectious disease

○③ Worry about being misunderstood and wrongly blamed

○④ Concern about lacking adequate first aid skills to succeed

○⑤ Believe it is unrelated to my responsibilities

○⑥ Hesitation to touch someone perceived as deceased

○⑦ Other:

2.13 If you were trained and mastered CPR, would you be willing to perform CPR on a stranger experiencing sudden cardiac arrest? (If you select "Unwilling," please proceed to Question 2.14)

○① Completely unwilling

○② Generally unwilling

○③ Somewhat willing

○④ Very willing

2.14 What is the primary reason for your unwillingness to perform CPR? (If you selected "Willing"

in Question 2.13, skip this question)

○① Find it unsanitary

○② Fear of contracting an infectious disease

○③ Worry about being misunderstood and wrongly blamed

○④ Concern about lacking adequate first aid skills to succeed

○⑤ Believe it is unrelated to my responsibilities

○⑥ Hesitation to touch someone perceived as deceased

○⑦ Other:

3.0 CPR knowledge awareness

3.1 If you encounter an unconscious injured person who has stopped breathing, what would you do first?

○① Perform chest compressions and rescue breaths immediately

○② Call the 120 emergency number immediately to seek help

○③ Panic and not know what to do

○④ Other: _______________

3.2 For an injured person with sudden cardiac arrest, irreversible brain damage due to hypoxia may occur unless CPR is performed within the "golden time." What is the "golden time"?

○① Within 4 minutes

○② Within 10 minutes

○③ Don’t know

3.3 If you find someone collapsed, how quickly should you determine whether they have suffered cardiac arrest to avoid delaying rescue?

○① Within 10 seconds

○② Around 30 seconds

○③ Don’t know

3.4 How do you check for consciousness in an adult?

○① Shout loudly and tap their shoulder gently

○② Shout loudly and shake their shoulders

○③ Don’t know

3.5 How should a person with sudden cardiac arrest be positioned?

○① Lying on their side

○② Lying flat on their back without a pillow

○③ Lying face down

○④ Don’t know

3.6 Where is the correct location for chest compressions in adults?

○① Left side of the chest

○② Center of the chest (mid-sternum, between the nipples)

○③ Right side of the chest

○④ Don’t know

3.7 What is the correct hand placement for adult chest compressions?

○① Overlapping hands with fingers pressing

○② Heel of one hand over the other, fingers lifted off the chest

○③ Don’t know

3.8 What is the correct arm posture during chest compressions?

○① Arms bent, using upper body and arm muscle strength

○② Arms straight, perpendicular to the chest, using upper body weight and arm strength

○③ Don’t know

3.9 What is the recommended depth for adult chest compressions?

○① Around 3 cm

○② 5–6 cm

○③ Don’t know

3.10 What is the recommended rate (per minute) for chest compressions?

○① Around 80 compressions

○② 100–120 compressions

○③ Don’t know

3.11 During rescue breaths, should you pinch the injured person's nose?

○① Yes

○② No

○③ Don't know

3.12 During rescue breaths, should you observe whether the chest rises?

○① Yes

○② No

○③ Don't know

3.13 How should rescue breaths be delivered?

○① Quick, forceful blows

○② Gentle, slow blows

○③ Don't know

3.14 In adult CPR, how many chest compressions should be followed by rescue breaths?

○① 15 compressions, then 2 breaths

○② 30 compressions, then 2 breaths

○③ Don't know

3.15 A CPR cycle typically involves how many compression-breath cycles before reassessing the injured person's condition?

○① 5 cycles

○② 10 cycles

○③ Don't know

3.16 How do you determine if CPR is successful? [Multiple choice]

□① Pupils constrict, with light reflex and eye movement

□② Skin color changes from cyanotic to rosy

□③ Restoration of major artery pulsations

□④ Return of spontaneous breathing

□⑤ Don't know

3.17 Under what circumstances should you stop CPR? [Multiple choice]

□① Medical professionals arrive and take over

□② The injured person regains spontaneous breathing and pulse

□③ A medical professional confirms death

□④ Don't know

4.0 Training channels and formats

4.1 What are your current sources for learning first aid knowledge? [Multiple choice]

□① Medical science books/magazines

□② Newspapers

□③ TV/radio broadcasts

□④ Online platforms

□⑤ Hearing from others

□⑥ Other, please specify: ______

4.2 Which training format do you find most convenient?

○① Lecture-based training

○② Hands-on practice sessions

○③ Watching videos/online self-study

○④ Senior health club activities

○⑤ Printed educational pamphlets

○⑥ Other: _______________

4.3 What is your preferred training schedule?

○① Complete training in a single session

○② Phased training based on content

○③ No preference

4.4 When would you prefer to attend training?

○① Weekdays (Monday–Friday)

○② Weekends or holidays

○③ Anytime

4.5 What is the ideal duration for each training session?

○① Less than 30 minutes

○② 30–60 minutes

○③ Over 60 minutes

4.6 How often should training be conducted?

○① 2–3 times per week

○② Once a week

○③ 2–3 times per month

○④ Once a month

○⑤ Other: _______________

4.7 What is your preferred training location?

○① Community hospital

○② Neighborhood committee office

○③ At home

○④ Other: _______________

Thank you for your participation and cooperation.
Please verify the above questions to make sure there are no empty items, thanks!

Questionnaire on health literacy related to cardiopulmonary resuscitation
(After training) and (6 months after training)

In order to actively mobilize the family members of patients with chronic diseases to participate in pre-hospital first aid, so that family members can effectively play the role of on-site first aid as the first witnesses, this project provides CPR knowledge and skills training to family members of chronic disease patients in community contracted families. In order to evaluate the effectiveness of the program and make the training of family health workers more targeted, we will conduct a survey on the CPR related situation of family members, and we will keep your personal information strictly confidential, please ensure the authenticity of the information filled in.

Research group of general hospitals and community health service systems cooperating to build a CPR training system for high-risk patients with sudden death.

August 6, 2021

Community Healthcare Center: ______________

1. Basic information

1.1 Name: _______________

1.2 Gender:

○①Male

○②Female

1.3 Age: ________

1.4 Education level:

○①Elementary school or below

○②Junior high school

○③Senior high school/ vocational school

○④University and above

1.5 Occupation:

○①Medical worker

○②Non-medical worker:

1.6 Annual family income (CNY, China Yuan; CNY/USD=0.14):

○①<50,000

○②50,000-100,000

○③>100,000

1.7 Marital status:

○①Unmarried

○②Married

○③Divorced

○④Widowed

1.8 Physical condition (multiple choice):

○①No chronic diseases

○②Hypertension

○③Diabetes

○④Coronary heart disease

○⑤Cerebrovascular disease

○⑥Mental disease

○⑦Other:

1.9 Do you have a family member who has got cardiovascular disease?

○①Yes

○②No

1.10 Whether there are any of the following risk factors for cardiovascular disease in the family members:

1.10.1 Elevated blood pressure（≥ 140/90 mmHg）

○①Yes

○②No

1.10.2 Elevated total cholesterol (TC) level（≥ 4.5 mmol/L）

○①Yes

○②No

1.10.3 Elevated low-density lipoprotein cholesterol（LDL-C）level（≥ 2.6 mmol/L）

○①Yes

○②No

1.10.4 Lowered high-density lipoprotein cholesterol（HDL-C）level（< 1.0 mmol/L）

○①Yes

○②No

1.10.5 Elevated fasting blood glucose（≥ 7.0 mmol/L）

○①Yes

○②No

1.10.6 Overweight or obese（BMI ≥ 24 kg/$m^2$ ）

○①Yes

○②No

1.10.7 Tobacco use

○①Yes: ___

○②No

2. Knowledge of CPR among family members who participates in the trainings

2.1 Have you heard of CPR before this survey?

○①Yes

○②No

2.2 Have you been trained in CPR before?

○①No

○②Yes, I was trained in CPR before.

2.3 Reasons for not having received CPR training previously (multiple choice).

□①Lack of awareness of its importance

□②Unable to find free training locations

□③Too busy with work or studies

□④No desire to attend training previously

□⑤Other:

2.4 How would you prefer to learn CPR?

○①On-site professional guidance

○②Video/audio learning

○③Learning via TV

○④Classroom-based learning

2.5 Have you encountered situations requiring emergency rescue?

○①No

○②Yes, encountered before

2.6 Do you think it is possible for patients with respiratory and/or cardiac arrest to be resuscitated?

○①Completely impossible

○②Generally impossible

○③Somewhat possible

○④Very possible

2.7 Necessity of learning CPR knowledge and skills for community residents.

○①Not necessary at all

○②Not necessary

○③Necessary

○④Very necessary

2.8 Do you believe you have the ability to learn CPR knowledge and skills?

○①Completely incapable

○②Generally incapable

○③Somewhat capable

○④Very capable

2.9 Do you have any interest in learning CPR knowledge and skills?

○① Completely uninterested

○② Generally uninterested

○③ Somewhat interested

○④ Very interested

2.10 Are you interested in participating in CPR training?

○ ①Completely uninterested

○ ②Generally uninterested

○ ③Somewhat interested

○ ④Very interested

2.11 If you were trained and mastered CPR, would you be willing to perform CPR on a family member or friend experiencing sudden cardiac arrest? (If you select "Unwilling," please proceed to Question 2.12)

○① Completely unwilling

○② Generally unwilling

○③ Somewhat willing

○④ Very willing

2.12 What is the primary reason for your unwillingness to perform CPR? (If you selected "Willing"

in Question 2.11, skip this question)

○① Find it unsanitary

○② Fear of contracting an infectious disease

○③ Worry about being misunderstood and wrongly blamed

○④ Concern about lacking adequate first aid skills to succeed

○⑤ Believe it is unrelated to my responsibilities

○⑥ Hesitation to touch someone perceived as deceased

○⑦ Other:

2.13 If you were trained and mastered CPR, would you be willing to perform CPR on a stranger experiencing sudden cardiac arrest? (If you select "Unwilling," please proceed to Question 2.14)

○① Completely unwilling

○② Generally unwilling

○③ Somewhat willing

○④ Very willing

2.14 What is the primary reason for your unwillingness to perform CPR? (If you selected "Willing" in Question 2.13, skip this question)

○① Find it unsanitary

○② Fear of contracting an infectious disease

○③ Worry about being misunderstood and wrongly blamed

○④ Concern about lacking adequate first aid skills to succeed

○⑤ Believe it is unrelated to my responsibilities

○⑥ Hesitation to touch someone perceived as deceased

○⑦ Other:

3. CPR knowledge awareness

3.1 If you encounter an unconscious injured person who has stopped breathing, what would you do first?

○① Perform chest compressions and rescue breaths immediately

○② Call the 120 emergency number immediately to seek help

○③ Panic and not know what to do

○④ Other: _______________

3.2 For an injured person with sudden cardiac arrest, irreversible brain damage due to hypoxia may occur unless CPR is performed within the "golden time." What is the "golden time"?

○① Within 4 minutes

○② Within 10 minutes

○③ Don’t know

3.3 If you find someone collapsed, how quickly should you determine whether they have suffered cardiac arrest to avoid delaying rescue?

○① Within 10 seconds

○② Around 30 seconds

○③ Don’t know

3.4 How do you check for consciousness in an adult?

○① Shout loudly and tap their shoulder gently

○② Shout loudly and shake their shoulders

○③ Don’t know

3.5 How should a person with sudden cardiac arrest be positioned?

○① Lying on their side

○② Lying flat on their back without a pillow

○③ Lying face down

○④ Don’t know

3.6 Where is the correct location for chest compressions in adults?

○① Left side of the chest

○② Center of the chest (mid-sternum, between the nipples)

○③ Right side of the chest

○④ Don’t know

3.7 What is the correct hand placement for adult chest compressions?

○① Overlapping hands with fingers pressing

○② Heel of one hand over the other, fingers lifted off the chest

○③ Don’t know

3.8 What is the correct arm posture during chest compressions?

○① Arms bent, using upper body and arm muscle strength

○② Arms straight, perpendicular to the chest, using upper body weight and arm strength

○③ Don’t know

3.9 What is the recommended depth for adult chest compressions?

○① Around 3 cm

○② 5–6 cm

○③ Don’t know

3.10 What is the recommended rate (per minute) for chest compressions?

○① Around 80 compressions

○② 100–120 compressions

○③ Don’t know

3.11 During rescue breaths, should you pinch the injured person's nose?

○① Yes

○② No

○③ Don’t know

3.12 During rescue breaths, should you observe whether the chest rises?

○① Yes

○② No

○③ Don’t know

3.13 How should rescue breaths be delivered?

○① Quick, forceful blows

○② Gentle, slow blows

○③ Don’t know

3.14 In adult CPR, how many chest compressions should be followed by rescue breaths?

○① 15 compressions, then 2 breaths

○② 30 compressions, then 2 breaths

○③ Don’t know

3.15 A CPR cycle typically involves how many compression-breath cycles before reassessing the injured person's condition?

○① 5 cycles

○② 10 cycles

○③ Don't know

3.16 How do you determine if CPR is successful? [Multiple choice]

□① Pupils constrict, with light reflex and eye movement

□② Skin color changes from cyanotic to rosy

□③ Restoration of major artery pulsations

□④ Return of spontaneous breathing

□⑤ Don't know

3.17 Under what circumstances should you stop CPR? [Multiple choice]

□① Medical professionals arrive and take over

□② The injured person regains spontaneous breathing and pulse

□③ A medical professional confirms death

□④ Don't know

4. Feedback on the Training Program

4.1 How satisfied are you with the course design of this CPR training program?

○① Very satisfied

○② Satisfied

○③ Neutral

○④ Somewhat dissatisfied

○⑤ Very dissatisfied

4.2 How satisfied are you with the trainers/instructors of this CPR training program?

○① Very satisfied

○② Satisfied

○③ Neutral

○④ Somewhat dissatisfied

○⑤ Very dissatisfied

4.3 How satisfied are you with the training content of this CPR training program?

○① Very satisfied

○② Satisfied

○③ Neutral

○④ Somewhat dissatisfied

○⑤ Very dissatisfied

4.4 How satisfied are you with the training methods of this CPR training program?

○① Very satisfied

○② Satisfied

○③ Neutral

○④ Somewhat dissatisfied

○⑤ Very dissatisfied

4.5 What is your overall satisfaction with this CPR training program?

○① Very satisfied

○② Satisfied

○③ Neutral

○④ Somewhat dissatisfied

○⑤ Very dissatisfied

Thank you for your participation and cooperation.

Please verify the above questions to make sure there are no empty items, thanks!
